# Mechanistic insights into non-immunosuppressive immunophilin ligands as potential antimalarial therapeutics

**DOI:** 10.1186/1475-2875-9-S2-P60

**Published:** 2010-10-20

**Authors:** Ho Sup Yoon, Reema Alag, Congbao Kang, Hong Ye, Masayo Kotaka, Insaf A Qureshi, Nagakumar Bharatham, Joon Shin, Zbynek Bozdech, Peter Preiser, Julien Lescar

**Affiliations:** 1 School of Biological Sciences, Nanyang Technological University, 60 Nanyang Drive, Singapore 637551

## Clinical background

**Figure 1:**
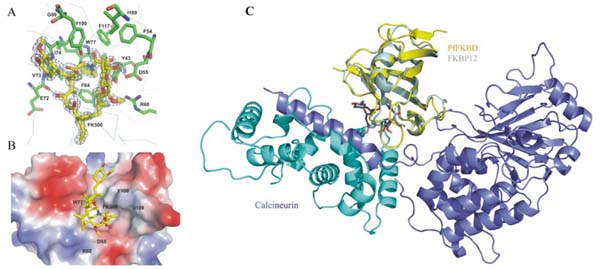
FK506 in complex with PvFKBD and ternary complex of FK506/PfFKBD/Calcineurin. (A) The electron density map of FK506 at the ligand binding site in PvFKBD. The residues near the FK506 are labeled and displayed as stick model in element colors. (B) Electrostatic surface representation of the residues near the FK506 binding sites in PvFKBD. Positive and negative charges are in blue and red, respectively. (C) Ternary complex model of FK506-bound PfFKBD and calcineurin. PfFKBD (yellow) was overlaid onto FKBP12 (pale cyan) of the FKBP12-FK506-calcineurin ternary complex in order to construct the ternary complex. FK506 is shown in sticks and calcineurin is shown in blue and cyan with its subunits A and B, respectively.

The immunosuppressive drug FK506 reveals an antimalarial activity. The mechanism of the drug action involves the molecular interaction with target proteins PfFKBP35 and PvFKBP35 from *P. falciparum* and *P. vivax,* respectively. Interestingly, non-immunosuppressive FK506 analogues also show antimalarial activities. This prompted us to attempt to develop potential small molecule antagonists that specifically target the parasite enzymes devoid of immunosuppressive activity. To this end, we have determined the three-dimensional structures of the FK506 binding domain (FKBD) of *P. falciparum* and *P. vivax* in unligand form and in complex with FK506.The structural studies reveal that the *Plasmodium* FKBPs have similar structural folds like the canonical FKBP folds (Figure 1 overleaf), providing mechanistic insights into PfFKBP35 and PvFKBP35 with possible routes for rational antimalarial drug design targeting the *Plasmodium* FKBPs.

## Conclusion

Our structure-guided drug screening efforts resulted in an identification of novel adamantanyl-based-antimalarial compound named Supradamal (SRA). SRA exhibits a nanomolar inhibitory activity in both peptidylprolyl cis-trans isomerase (PPIase) assay and growth of *P. falciparum* cultured in human erythrocytes. In particular, SRA appears to inhibit the trophozoite development.
